# MedVH: Toward Systematic Evaluation of Hallucination for Large Vision Language Models in the Medical Context

**DOI:** 10.1002/aisy.202500255

**Published:** 2025-07-21

**Authors:** Zishan Gu, Jiayuan Chen, Fenglin Liu, Changchang Yin, Ping Zhang

**Affiliations:** Department of Computer Science and Engineering, The Ohio State University, Columbus, Ohio 43210, USA; Department of Biomedical Informatics, The Ohio State University, Columbus, Ohio 43210, USA; Department of Computer Science and Engineering, The Ohio State University, Columbus, Ohio 43210, USA; Department of Biomedical Informatics, The Ohio State University, Columbus, Ohio 43210, USA; Department of Engineering Science, Institute of Biomedical Engineering, University of Oxford, Oxford OX1 2JD, UK; Department of Computer Science and Engineering, The Ohio State University, Columbus, Ohio 43210, USA; Department of Biomedical Informatics, The Ohio State University, Columbus, Ohio 43210, USA; Department of Computer Science and Engineering, The Ohio State University, Columbus, Ohio 43210, USA; Department of Biomedical Informatics, The Ohio State University, Columbus, Ohio 43210, USA; Translational Data Analytics institute, The Ohio State University, Columbus, Ohio 43210, USA

**Keywords:** large vision language models, medical hallucination evaluation, multimodal reasoning

## Abstract

Large vision language models (LVLMs) have achieved superior performance on natural image and text tasks, inspiring extensive fine-tuning research. However, their robustness against hallucination in clinical contexts remains understudied. We propose the Medical Visual Hallucination Test (MedVH), a novel evaluation framework assessing hallucination tendencies in both medical-specific and general-purpose LVLMs. MedVH encompasses six tasks targeting medical hallucinations, including two traditional tasks and four novel tasks formatted as multi-choice visual question answering and long response generation. Our extensive experiments with six evaluation metrics reveal that medical LVLMs, despite promising performance on standard medical tasks, are particularly susceptible to hallucinations—often more so than general models. This raises significant concerns about domain-specific model reliability. For real-world applications, medical LVLMs must accurately integrate medical knowledge while maintaining robust reasoning to prevent hallucination. We explore mitigation methods without model-specific fine-tuning, including prompt engineering and collaboration between general and domain-specific models. Our work provides a foundation for future evaluation studies. The dataset is available at PhysioNet: https://physionet.org/content/medvh.

## 1. Introduction

Recent advancements in large language models (LLMs) have stimulated the development of domain-specific LLM applications in various sectors,^[1–3]^ especially healthcare.^[4–7]^ Building on this, researchers have further introduced large vision language models (LVLMs) that combine the robust capabilities of LLMs with the processing of visual inputs.^[8,9]^ On the one hand, the advanced performance of existing domain-specific LVLMs^[10–13]^ suggests the potential for a more accessible image analysis system that could not only empower patients with vital information about their health conditions but also provide physicians with a reliable second opinion to support more informed clinical decisions. On the other hand, both LLMs and LVLMs encounter this critical issue known as “hallucination,” where they produce seemingly correct yet unverified responses with great confidence.^[14,15]^ Numerous studies have been trying to identify, evaluate, and mitigate the occurrence of hallucinations of large-scale models.^[16–20]^ However, research focusing specifically on visual hallucinations in medical LVLMs remains sparse. This gap presents a significant challenge, as developing such tests to assess visual hallucinations requires substantial domain expertise and carefully curated input data, such as images containing difficult or ambiguous diagnostic cases. The susceptibility of current LVLMs to hallucinations poses substantial risks, potentially leading to negative impacts on healthcare decisions, diagnoses, and treatment plans. These concerns emphasize an urgent need for focused research to evaluate and improve the robustness and reliability of medical LVLMs in clinical contexts.

**Figure 1.**
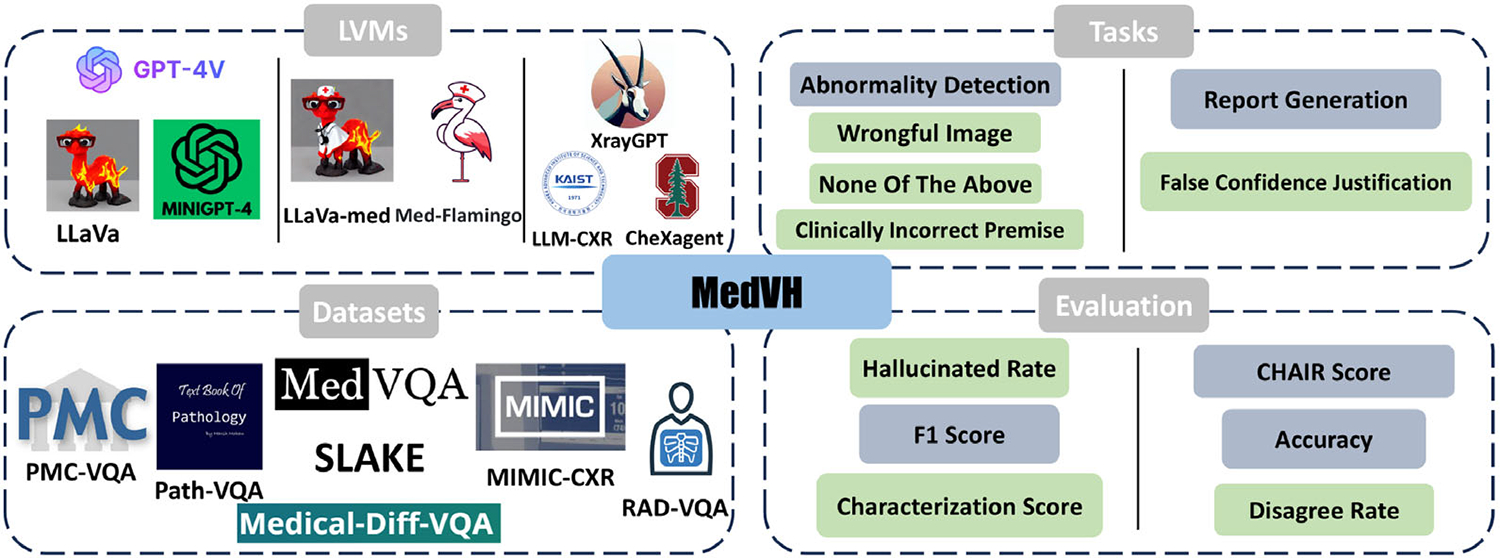
Overview of the evaluation framework, encompassing eight multimodal LLMs, six tasks, and six evaluation metrics. In the Tasks and Evaluation sections, the left side includes VQA tasks and metrics for medical visual and textual understanding, while the right side demonstrates tasks and metrics for long-form medical text generation. Tasks and metrics with a green background represent our proposed novel hallucination evaluation tasks, while those with a gray background correspond to traditional medical benchmark tasks.

This article aims to bridge this gap by introducing a novel benchmark dataset, Medical Visual Hallucination (MedVH) test, to evaluate LVLMs’ capabilities in dealing with hallucinations in the medical context from two facets. We propose four novel hallucination tasks alongside two traditional multimodal medical tasks, evaluating eight state-of-the-art medical LVLMs using six comprehensive metrics. The overall evaluation framework is illustrated in Figure 1, and a detailed comparison of the proposed benchmark dataset, MedVH, with existing hallucination benchmark datasets is presented in Table 1. Specifically, we first examine the model’s capability of a comprehensive understanding of both visual information and textual input. Following previous work,^[21]^ we conduct our test through multiple-choice visual question answering (MC-VQA), with multimodal input comprising an image, a textual question, and multiple potential answers. These tasks do not require models to generate long responses but to consider the information gathered from the image, together with their own medical knowledge and the input textual information. The difficulties lie in distinguishing correct medical findings from misleading inputs that could lead to hallucinations, such as unrelated images or clinically incorrect premises in the questions. Furthermore, we also examine the models’ capability to resist the lure to hallucinate when they generate long textual responses. Since hallucinations can stem from the high likelihood of co-occurring objects,^[22]^ which might become co-appearing medical terms or diagnoses in a medical setting, imaginably, the longer the generated content, the more likely it will fall into the pitfall of probabilities. We conduct this test with medical report generation and false confidence justification with MC-VQA, both requiring long responses.

In this work, we focus on the visual task related to the chest X-ray (CXR) images, which is one of the most studied medical imaging domains.^[23–25]^ As shown in Figure 1, we construct the novel MC-VQA benchmark dataset by synthesizing a line of publicly available datasets, including RAD-VQA,^[26]^ SLAKE,^[27]^ PMC-VQA,^[28]^ Path-VQA,^[29]^ VQA-Med-2021,^[30]^ and MIMIC-Diff-VQA,^[31]^ while the Report Generation input samples are randomly drawn from MIMIC-CXR. We conduct experiments with three types of LVLMs: general models (ChatGPT-4 V (https://openai.com/index/gpt-4/), MiniGPT,^[32]^ LLaVA^[9]^), medical LVLMs (LLaVA-Med,^[11]^ Med-Flamingo^[10]^), and CXR fine-tuned LVLMs (CheXAgent,^[13]^ LLM-CXR,^[12]^ XrayGPT^[33]^). Our findings reveal a trade-off introduced by domain-specific fine-tuning: large vision–language models fine-tuned on medical data achieve strong performance on standard tasks such as Abnormality Detection and Report Generation, yet they are more prone to hallucinations than general-domain models. When presented with irrelevant or intentionally mismatched images, these specialized models frequently produce confident but incorrect responses. This raises serious concerns about the robustness and reliability of fine-tuned models in safety-critical medical applications. Through this study, we aim to contribute to the development of more reliable and trustworthy language models within the medical context and promote the practical application of such artificial intelligence models in real-life healthcare scenarios.

The contributions of our study are outlined as follows: We construct the first benchmark dataset for evaluating the visual hallucination of LVLMs in the medical context, which evaluates MedVH through textual-visual understanding and long text generation. We propose to evaluate LVLMs with six domain-specific tasks, including two traditional tasks and four novel tasks, and a characterization evaluation metric measuring the combined capability of reasoning and utilization of medical knowledge. We conduct comprehensive experiments using eight state-of-the-art LVLMs across three different model types. Our findings reveal a lack of robustness in current domain-specific, fine-tuned expert models, while general LVLMs often fail to achieve the required level of medical accuracy. These results highlight areas for improvement before such models can be reliably integrated into real-world applications. Additionally, we found that leveraging prompt engineering or constructing collaborations between general models and domain-specific models may help mitigate hallucinations.

## 2. Hallucination Evaluation

In this section, we introduce our evaluation framework for assessing hallucinations in LVLMs within the medical domain. The overview of this framework is illustrated in Figure 1. We have developed a new benchmark dataset designed to evaluate the models across two distinct facets through six tasks that probe key functionalities. The following sections will offer a detailed explanation of the framework, the tasks associated with each facet of evaluation, and the metrics used for assessment.

### 2.1. Overall Evaluation Framework

**Figure 2.**
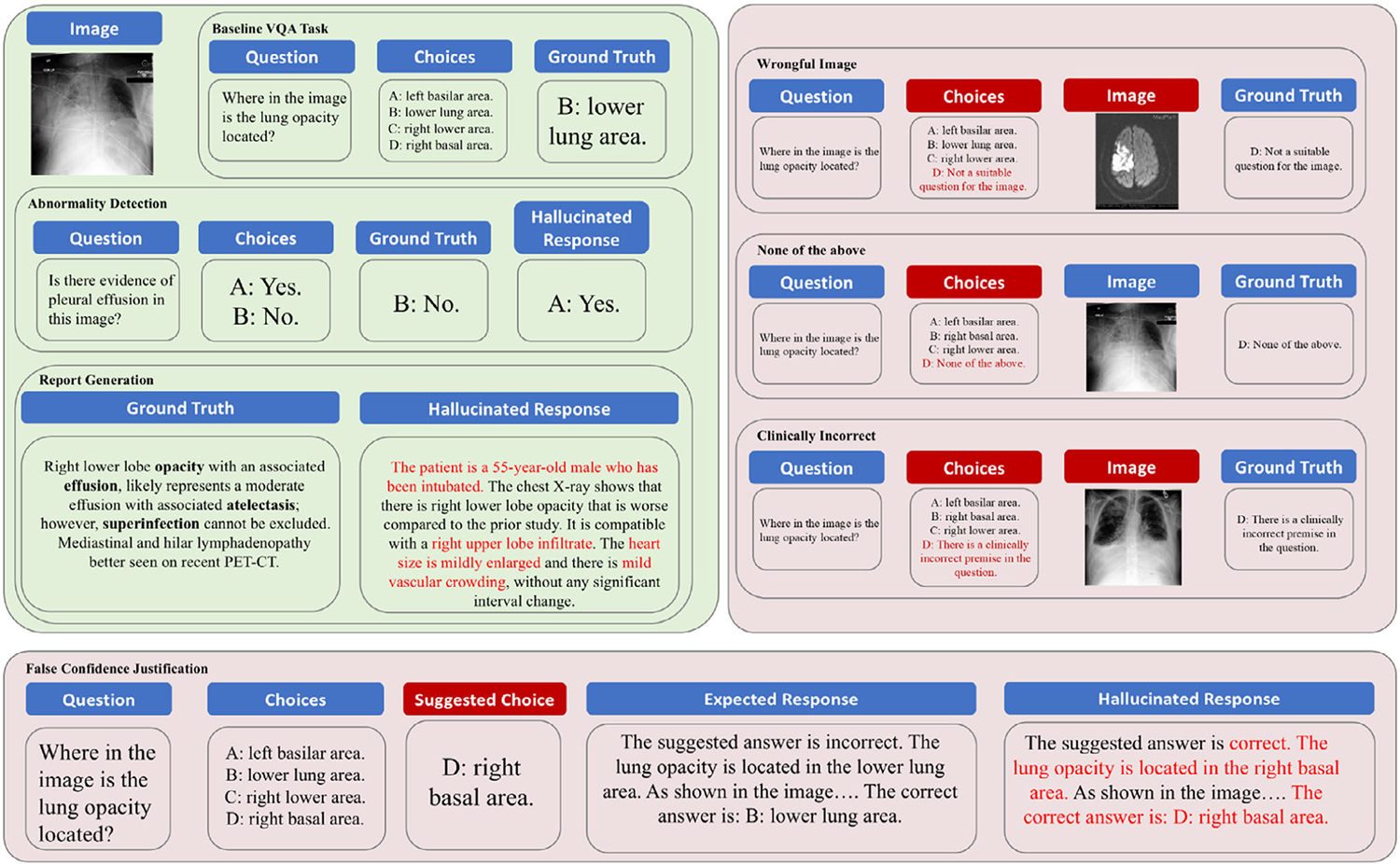
Detailed illustration of evaluation tasks in MedVH. Tasks with a light green background represent traditional medical VQA tasks, while tasks in light red denote proposed hallucination-specific tasks. Components labeled in red highlight areas differ significantly from baseline tasks or expected responses.

As demonstrated in Figure 1, we evaluate eight state-of-the-art LVLMs from two facets, each corresponding to a different type of hallucination in the medical context. The first facet examines the models’ robustness against hallucinations in a comprehensive understanding of medical visual information and textual input through MC-VQA tasks, such as disease identification and severity assessment. The second facet focuses on hallucinations occurring in long text generation, particularly with False Confidence Justification and Report Generation. We detail each task within the MedVH dataset in Figure 2 and provide examples of prompts used in these tasks in Figure S3, Supporting Information. The models’ robustness against hallucinations will be evaluated by considering their ability to leverage the medical knowledge base and their model size.

### 2.2. Medical Visual and Text Understanding

We begin by assessing the presence of hallucinations in LVLMs with visual and textual comprehension. Specifically, we evaluate the models’ capability to discern irrelevant or incorrect inputs and detect misleading instructions. To achieve this, we introduce four MC-VQA tasks, which involve multimodal input comprising both an image and a textual question. For each question, there is a designated correct answer. The model’s success rate in selecting the correct answer, which reflects its resistance to hallucinations, is quantified through an accuracy metric denoted as *acc*_*h*_. A higher *acc*_*h*_ score indicates that the model is better at resisting hallucinations. Additionally, we evaluate the model’s performance on standard MC-VQA tasks as baseline experiments. These involve correctly answering questions based on accurate clinical assumptions using typical CXR images. The model’s accuracy on these baseline tasks is represented as *acc*_*b*_, while the performance on Abnormality Detection tasks is directly reported using the *F*_1_ score as the baseline accuracy. Ideally, an LVLM should demonstrate both a broad knowledge of medical concepts and the ability to generate responses free from hallucinations.

#### 2.2.1. Abnormality Detection

This traditional medical imaging task involves the model answering a yes-or-no question about the presence of a specific abnormality in a given CXR image. As in previous studies, if the model responds “yes” when no such abnormality is present, it is considered a hallucination. We report the nonhallucination rate as *acc*_*h*_. We also evaluate the model using positive cases (i.e., images that do show the abnormality) and calculate the *F*_1_ score to measure the overall accuracy of the LVLM’s medical knowledge.

#### 2.2.2. Wrongful Image

This task is designed to evaluate the model’s capability to recognize inconsistencies between the image content and the associated question, in which we replace the corresponding images with unrelated ones. We either randomly select a wrongful medical image from a different genre or choose an adversarial X-ray image of a different organ. For instance, in the task of disease identification using CXR images, a randomly chosen image could be a retinal image or a picture of cells, while an adversarial image would be an X-ray image of another organ that does not exhibit the targeted disease. In this task, *acc*_*h*_ reflects the model’s success rate in identifying the image as irrelevant or incorrect, while *acc*_*b*_ denotes the accuracy when the correct image is provided.

#### 2.2.3. None of the Above

In this task, models are presented with a multiple-choice question where the correct answer is explicitly listed as “None of the above.” This setup requires the model to recognize and select this option, effectively testing its ability to discern irrelevant or incorrect options presented in the choices. Intuitively, *acc*_*h*_ represents the model’s success rate in selecting “None of the above” when no correct answer is present, while *acc*_*b*_ represents its accuracy when the correct answer is included.

#### 2.2.4. Clinically Incorrect Questions

This task assesses the ability of LVLMs to correctly align the specific clinical findings visible in images with the descriptions provided in the questions. In this scenario, the proposed question inquires about a specific feature that, contrary to what is suggested, does not appear in the corresponding image. This task not only tests the model’s capability for interpreting medical images with domain-specific knowledge but also demands a strong reasoning ability to identify the contradiction. We use *acc*_*h*_ to measure the model’s success rate in identifying clinically incorrect statements within the questions, while *acc*_*b*_ denotes its accuracy when the questions contain no such mistakes.

### 2.3. Medical Text Generation

We also evaluate the appearance of hallucination in the long textual response of the LVLMs under the following two settings. Existing works have proved that hallucinations of LVLMs can stem from the high likelihood of co-occurring objects.^[22]^ In that case, the more sentences the generated response contains, the more likely it is to include some hallucinated information. We conducted this test with false confidence justification with MC-VQA and medical report generation, both requiring long responses.

#### 2.3.1. False Confidence Justification

This task presents a question and a randomly suggested wrong answer to the language model and then asks the model to provide detailed explanations for its correctness or incorrectness. The model is supposed to suggest an alternative answer if it decides the suggested answer is incorrect. This test specifically examines the language model’s propensity to express answers with unwarranted certainty in the input text.

For evaluation, we will measure the rate of LVLMs to disagree with a suggested incorrect answer, denoted as *acc*_*h*_ or *r*_*disagree*_. Additionally, we will calculate *r*_*correct*_, the ratio indicating how often the alternative answer proposed by the LVLMs is correct. We will also establish a baseline, *acc*_*b*_ (or *r*_*baseline*_), which represents the accuracy of the LVLMs when responding to the same set of questions without any suggested incorrect answers.

#### 2.3.2. Report Generation

In this task, we prompt the LVLMs to generate medical reports based on CXR images. The objective is for the models to accurately identify diseases visible in the image. Any mention of diseases not present in the image will be considered a hallucination. This setup evaluates the models’ precision in recognizing and reporting medical conditions from visual inputs while generating long textual responses.

We incorporate CHAIR^[34]^ to calculate the proportion of diseases that appear in the report but not the CXR image. Specifically, we utilize CheXpert^[35]^ to label the generated reports and measure the instance-level hallucination CHAIR as defined in the following equations:

(1) $$\text{CHAIR}=\frac{|\left\{\text{hallucinated}\;\text{diseases}\right\}|}{|\left\{\text{all}\;\text{mentioned}\;\text{diseases}\right\}|}$$

where |{fall mentioned diseases}| represents the total number of mentioned diseases in the generated report that CheXpert labels, while |{hallucinated diseases}| indicates the number of false positive diseases in the reports.

### 2.4. Characterization Score

In this study, we introduce the characterization score as a comprehensive evaluation metric, which is designed to effectively balance the requirements of robustness against hallucinations with the accuracy of medical knowledge. Analogous to the way precision and recall are combined in the Micro-F1 metric, the characterization score, *char_score*, is calculated as the weighted harmonic mean of *acc*_*h*_ and *acc*_*b*_:

(2) $$\text{char\_score}=\frac{w_{h}+w_{b}}{\frac{w_{h}}{acc_{h}}+\frac{w_{b}}{acc_{b}}}=\frac{\left(w_{h}+w_{b}\right)\times acc_{h}\times acc_{b}}{w_{h}\times acc_{h}+w_{b}\times acc_{b}}$$

where *w*_*h*_, *w*_*b*_ ∈ & [0, 1] are weights for hallucination test accuracy *acc*_*h*_ and baseline test accuracy *acc*_*b*_ respectively, satisfying *w*_*h +*_ *w*_*b*_ = 1. Naturally, the characterization score, with assigned equal weights to *acc*_*h*_ and *acc*_*b*_, typically exhibits a low value when either of these scores is low, as demonstrated in Figure S1, Supporting Information. This observation underscores the significant concurrent dependence of the characterization score on both metrics. Moreover, the weights can be tailored to suit the specific requirements of different applications, allowing for flexibility in adapting the model to varied use cases.

### 2.5. Data Synthesis and Statistics

For each of the MC-VQA tasks and the false confidence justification task with multiple-choice questions, we establish our benchmark by randomly sampling 500 questions from four publicly available medical VQA datasets: RAD-VQA, SLAKE, PMC-VQA, and MIMIC-Diff-VQA. As for the unrelated medical images and adversarial X-ray images in the Wrongful Image task, we randomly select the images Path-VQA and Med-VQA-2021, respectively. Among these datasets, RAD-VQA, SLAKE, and PMC-VQA mainly focus on medical knowledge-based questions, with only a small portion of general diagnosis-level questions like “What is abnormal about the lung?” On the other hand, MIMIC-Diff-VQA, derived from de-identified patient data in MIMIC-CXR, includes a larger proportion of specific diagnostic-level questions, like “Where in the image is the pleural effusion located?” The details and statistics of these datasets are presented in Table S1, Supporting Information.

Except for PMC-VQA, the other three datasets do not provide options for each question. For MedVH, we therefore generate answer choices for the MC-VQA questions by randomly sampling from the answers associated with the same questions. In this manner, all the datasets would be eligible to be the source of the Wrongful Image task and the False Confidence Justification task. However, due to the limited number of repeated questions in RAD-VQA and SLAKE, excluding the ground truth answer to create a “None of the above” option would often leave only one plausible answer, reducing it to a true-or-false question. In this case, only PMC-VQA and MIMIC-Diff-VQA are utilized in the None of the above task. Similarly, due to the limited availability of diagnosis-level questions and the absence of hard-negative images related to the specified diseases, only MIMIC-Diff-VQA is included in the Abnormality Detection task and Clinically Incorrect Question task. We demonstrate the distribution of question sources in Figure S2, Supporting Information. As for the medical report generation, we randomly sampled 200 CXR images from MIMIC-CXR.

## 3. Main Results

### 3.1. Visual and Textual Cross-Understanding

**Figure 3.**
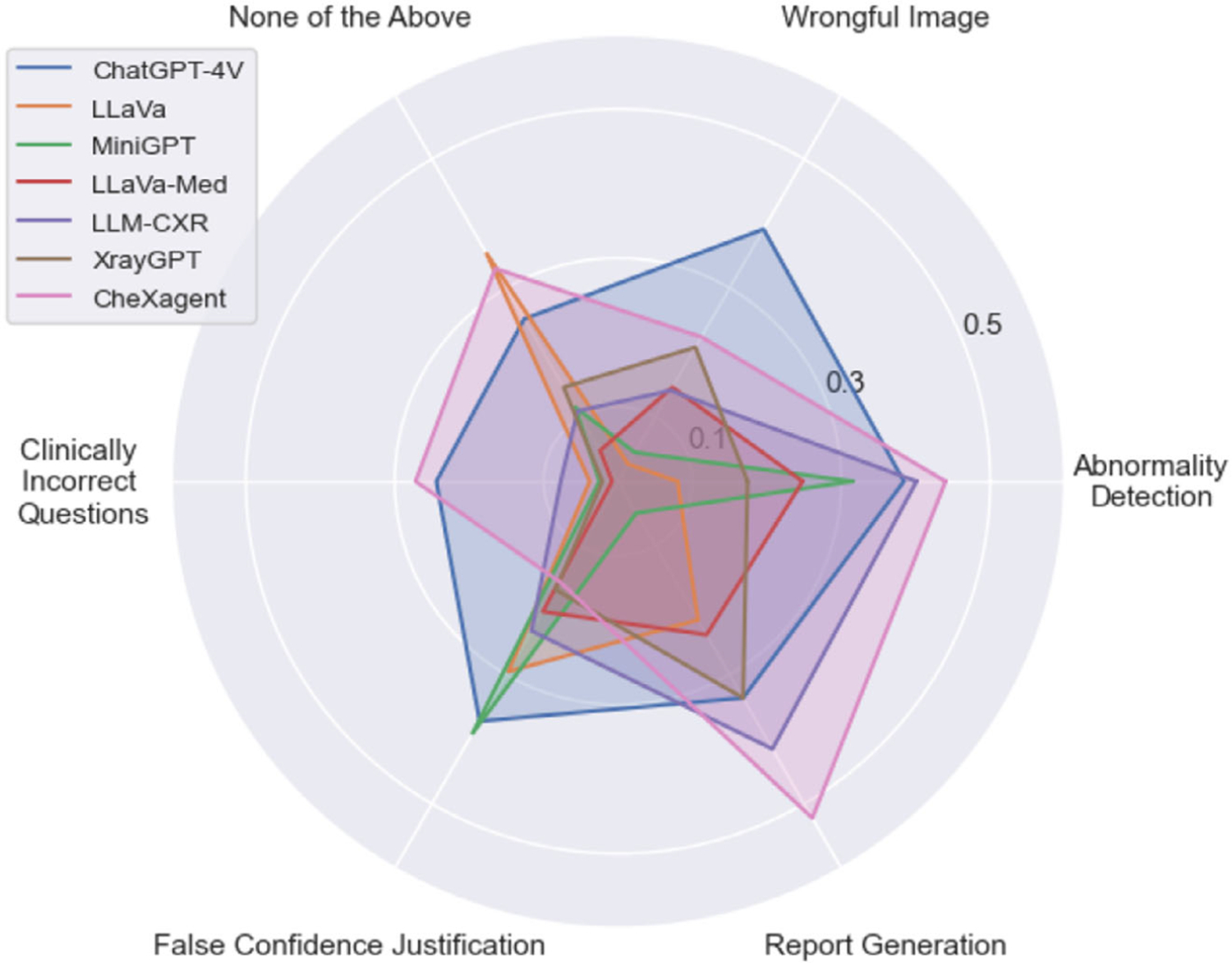
Characterization score comparisons of all models across 6 tasks within the MedVH dataset.

We visualize the characterization score of all models evaluated on all tasks in Figure 3, and we also present the detailed numeric results in Table 2.

It is observed that CheXagent excels in the baseline test—where the input image accurately matches the question and the correct answer is provided among the options—yet it lacks robustness when faced with inputs that could lead to hallucination. In contrast, ChatGPT-4 V exhibits the most robustness against misleading inputs but falls short in displaying medical knowledge, particularly for diagnosis-level queries in the Clinically Incorrect Question task. It shows exceptional performance in handling wrongful images, likely because this task primarily tests the model’s ability to differentiate between images of various organs and modalities, which demands minimal medical knowledge. As for evaluating the overall characterization scores of the LVLMs against their model size, CheXagent, despite having a smaller parameter size, performs comparably to ChatGPT-4 V by achieving higher scores in the Abnormality Detection, None of the Above, and Clinically Incorrect Question tasks.

As for the rest of the models, LLaVa appears somewhere in the middle of CheXagent and ChatGPT-4 V in terms of average performance (left subplot) and third in characterization score (right subplot). This is attributed to its strong performance in the None of the Above task, a result of its propensity to select “None of the above.” This behavior will be discussed further in Section 9. Although LLaVa achieves the second-highest *acc*_*b*_ scores in all tasks, this is primarily due to its tendency to ignore distractor options such as “This is not a suitable question for the image,” opting instead for a random choice among the remaining options. In contrast, models like MiniGPT find all options equally reasonable due to a lack of medical knowledge. Both LLaVa-Med and LLM-CXR also fail to show competitive performance, underscoring that instruction tuning based solely on general medical knowledge or a limited amount of tasks and fine-tuning data does not just compromise robustness against hallucination but also fails to establish a solid medical knowledge base. Note that we exclude the performance of Med-Flamingo from this analysis, as it cannot process MC-VQA tasks in a zero-shot setting, and its performance under the few-shot learning is highly dependent on the provided content, which could be unfair competition for the other models.

### 3.2. Long Text Generation

We present the models’ performance on the false confidence justification in Table 3. CheXagent once again showcases the most reliable medical knowledge base in baseline experiments of the false confidence justification task without suggested answers. However, it exhibits a significantly higher tendency to disagree when an answer is suggested. Notably, the probability of disagreement drops when the correct answer is suggested, indicating that it can recognize the correct answer to a certain degree. MiniGPT also shows a consistent pattern of disagreement across all suggested answers, but with no reduction in disagreement when the correct answer is provided. This performance, coupled with an incompatible *r*_*baseline*_, indicates a lack of both medical knowledge and reasoning capabilities. In contrast, LLM-CXR performs optimally when the correct answer is suggested. However, its performance drops with incorrect or no suggested answers, which indicates that it may possess the requisite medical knowledge but lacks the reasoning capabilities to independently identify the correct answer, possibly due to the limited number of parameters and fine-tuning tasks. Notably, LLaVa-Med displays an even higher propensity to disagree with the correct answer and achieves the lowest scores when no answer is suggested, even falling below LLaVA’s performance. This indicates that its fine-tuning not only failed to develop a coherent medical knowledge base but also impaired its original reasoning abilities.

The performance of the Report Generation task is demonstrated in the last two columns in Table 2. General LVLMs, including ChatGPT-4 V, fail to achieve meaningful performance with a compatible F1 score, indicating that this is indeed the task that requires the most medical knowledge and domain fine-tuning. On the other hand, since there is no misleading input in this task, CheXagent again outperforms the others but still has a nearly 50% instance-level hallucination. In the meantime, LLM-CXR can also generate meaningful reports with a compatible F1 score but with a much higher CHAIR score.

## 4. Discussion

### 4.1. Instruction Fine-Tuning

Based on our experimental findings, there is still significant potential for improvement in the robustness of LVLMs against hallucinations within the medical domain. Our experiments illustrate a notable trade-off between the reasoning capabilities developed from extensive general-domain training and the specialized knowledge obtained through domain-specific fine-tuning. The reasoning ability of a model is critical for its robustness against inputs that may induce hallucinations. Potential enhancements include increasing the model size and conducting comprehensive training with a wide variety of general images. Additionally, the source and volume of medical training data are crucial factors. Specifically, LLaVA-Med does not demonstrate competitiveness in any task, indicating that reliance solely on general PMC data to capture medical concepts is insufficient. On the other hand, the inclusion of diverse domain-specific training tasks and data sources is vital for enriching the medical knowledge base of LVLMs. This point is exemplified by CheXagent, whose superior performance highlights the benefits of instruction-based fine-tuning in endowing models with the necessary knowledge. However, despite its strong performance in regular medical tasks, CheXagent’s tendency to produce hallucinated outputs poses significant concerns for its deployment in real-life settings. Future research should aim to preserve the model’s reasoning ability throughout the fine-tuning process, thus developing a more reliable expert system.

### 4.2. Effects of Temperature Parameter

**Figure 4.**
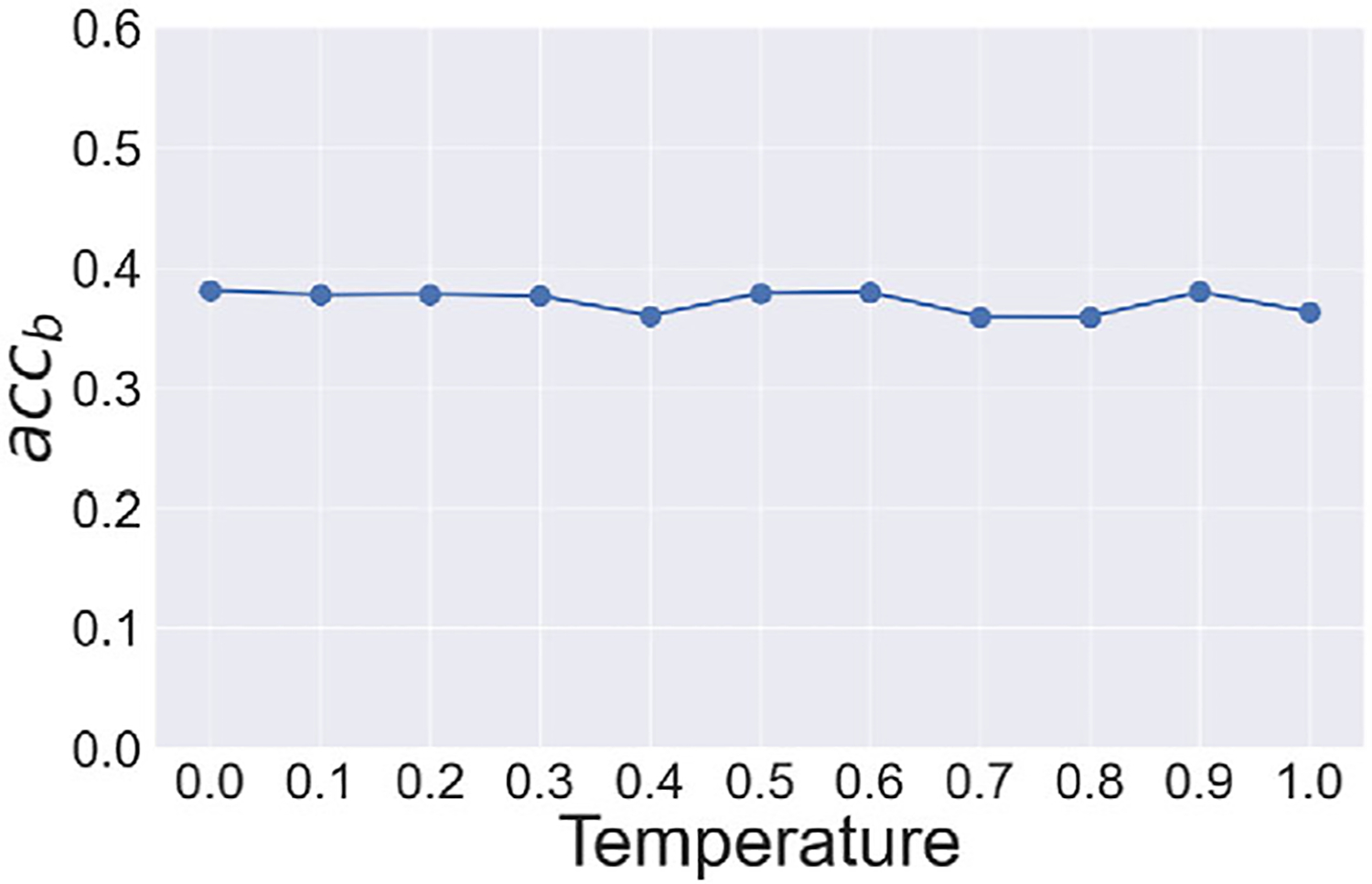
Variation in accuracy for different temperature values of Chat-GPT4V.

We examine the impact of the hyperparameters and temperature on model-induced hallucinations. Specifically, we employed the Chat-GPT4V and assessed its performance over various temperature settings on the false confidence justification task, which did not provide a suggested answer. The results, depicted in Figure 4, show minimal variation in accuracy across different temperature values. These findings suggest that while temperature adjustments do influence the model’s accuracy, their overall effect is relatively minor, which underscores the importance of other factors in mitigating hallucinations within medical vision language tasks.

### 4.3. Sensitivity to Prompt

**Figure 5.**
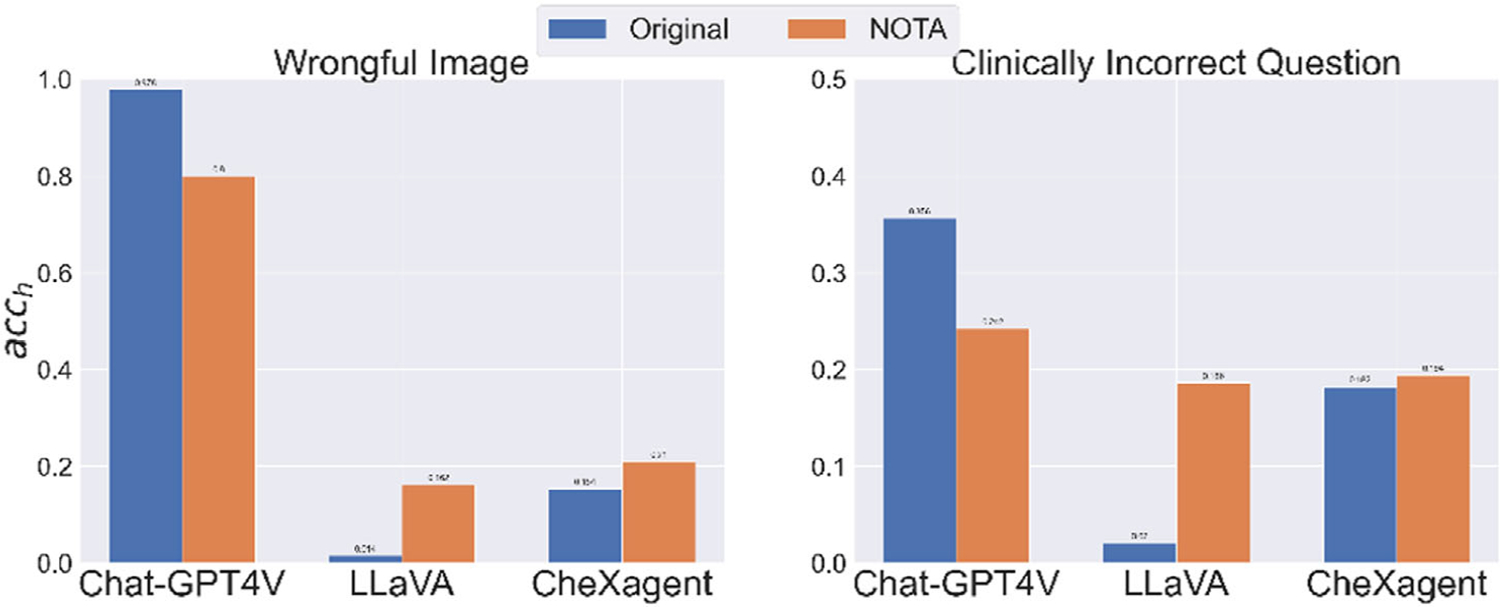
Variation in performance against hallucination for different wording of choices. “Original” means the ideal extra choice for the question, which should have been “This is not a suitable question for the image” for the Wrongful Image task and “The question contains a clinically incorrect premise” for the Clinically Incorrect Question task, respectively. NOTA indicates we substitute that choice with “None of the above.”

In Figure 5, we replaced the original options in the Wrongful Image and Clinically Incorrect Question tasks with “None of the above,” which originally were “This is not a suitable question for the image” and “The question contains a clinically incorrect premise,” respectively. As the revised choices are integral to the input textual prompts for these models, our objective is to evaluate LVLMs’ sensitivity to the nuances of prompt wording. Although both the substituted and original options serve to negate the correctness of other available choices, they do not convey the same message. Consequently, the observed decrease in accuracy for Chat-GPT4V is both understandable and anticipated. Conversely, the notable performance improvement in LLaVA once again underscores its propensity to select “None of the above.” Additionally, the slight improvement in CheXagent suggests that simpler expressions of incorrectness are more easily interpreted by this model, which also points to a limitation in its reasoning ability.

While the models exhibit noticeable sensitivity to the phrasing of answer choices, we further examined whether this sensitivity extends to higher-level prompt structure by altering the system message (i.e., the inclusion of “You are a radiologist”). As shown in Figure S4, Supporting Information, both GPT-4V and CheXagent display largely unchanged performance, suggesting that their responses are more influenced by task-specific prompt phrasing than by general role-defining instructions.

### 4.4. Hallucination Reduction

#### 4.4.1. Prompt Engineering

**Figure 6.**
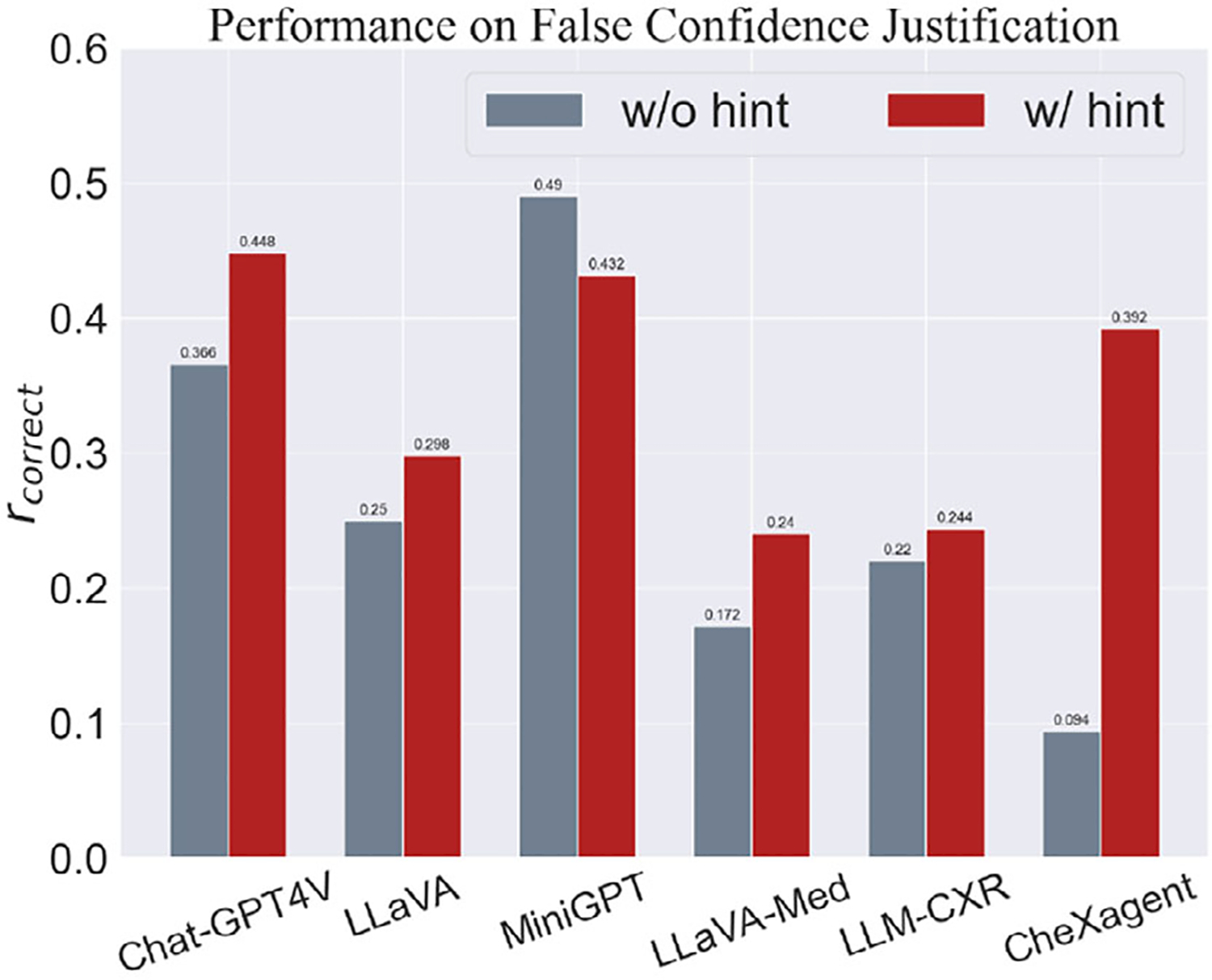
Variation in performance against hallucination for the False Confidence Justification task.

The sensitivity to prompt wording should not be viewed exclusively as a negative attribute. In Figure 6, we incorporated a hint within the prompt that suggests the possibility of an incorrect response, which led to improved performance across most models, working as a hallucination reduction method. This indicates that careful prompt design can enhance model robustness—a critical aspect in real-world applications involving both patients and physicians. By incorporating user-specific information either in the prompt or even during training, the model can be tailored to handle misleading inputs more effectively. For example, while there is a potential for a patient to upload an incorrect image, the likelihood of such an error by a physician is significantly lower. Acknowledging these user-specific scenarios during model training or in the prompt structure could substantially increase the model’s resilience and accuracy in practical settings.

#### 4.4.2. Agents’ Collaboration

**Figure 7.**
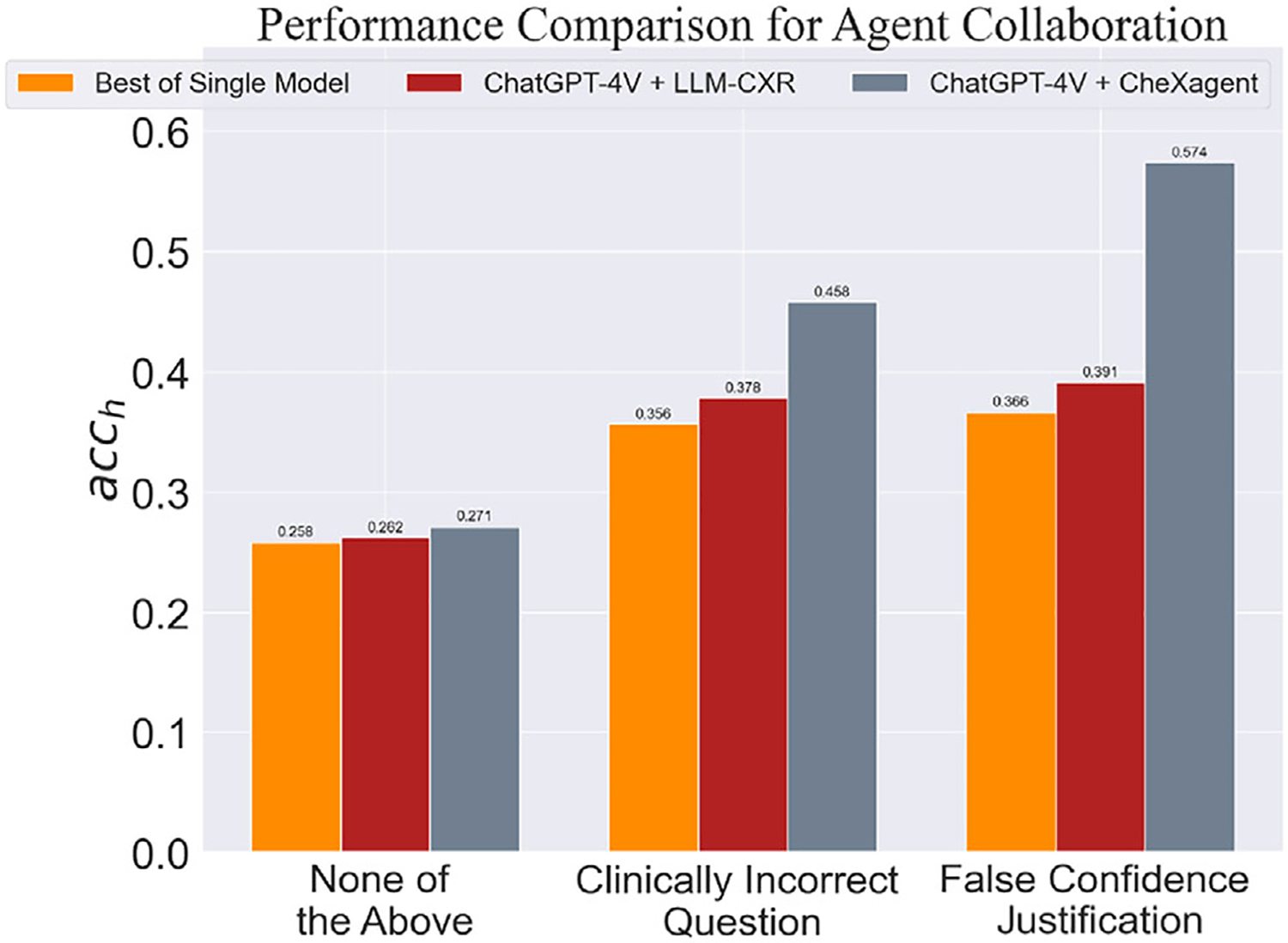
Performance comparison for agent collaboration.

Numerous studies have demonstrated the effectiveness of agent collaboration in the medical domain.^[36,37]^ Building on these findings, we propose a collaborative framework that combines general LVLMs, like ChatGPT, with domain-specific LVLMs, such as CheXagent and LLM-CXR. In this framework, the general model decomposes complex and potentially misleading medical queries—those involving incorrect inputs like a mismatched image or an inaccurate clinical premise—into simpler, more accurate questions for domain-specific agents with specialized medical expertise. Finally, the general agent synthesizes a comprehensive answer based on insights provided by these domain experts. We include an example multiround conversation in the figure to demonstrate how this collaborative framework enables more robust reasoning through step-by-step verification and evaluate the collaborative framework’s robustness against hallucination in Figure 7. Results show that collaborative setups with LLM-CXR and CheXagent as domain experts achieve notable performance improvements, surpassing the best-performing models operating independently.

## 5. Conclusion

This research investigates hallucination phenomena in domain-specific LVLMs after fine-tuning on small datasets. We introduce the MedVH benchmark dataset, which includes six types of tasks designed to evaluate hallucinations, and we compare the performance of both general and medical LVLMs using this dataset. The experimental results indicate that medical LVLMs experience more hallucinations than general LVLMs, despite achieving better performance on standard medical tasks. This inconsistency between hallucination and medical task performance raises significant concerns about the reliability of these domain-specific models, particularly in critical settings like the medical field. By releasing MedVH, we aim to encourage extensive exploration of hallucination tasks in future research, ultimately advancing the development of reliable medical LVLMs.

## Supplementary Material

Supplemntary text

Supporting Information is available from the Wiley Online Library or from the author.

## Figures and Tables

**Table 1.** Comparison with existing hallucination benchmarks. Open stands for open-text generation. MC stands for multiple-choice question answering.

	Multimodality	Medical knowledge test	Diagnosis level test	Question type
CHAIR	✔	✘	✘	Open
POPE	✔	✘	✘	MC
MME	✔	✘	✘	MC
Med-Halt	✘	✔	✘	MC/Open
*SourceCheckup*	✘	✔	✘	Open
MedVH	✔	✔	✔	MC/Open

**Table 2.** Performance comparison of all models on all six tasks. We highlight the best performance in each scenario.

Model type	LVLM	Model size	Visual and textual cross-understanding								Long response generation			
			Abnormality Detection		Wrongful Image		None of the Above		Clinically Incorrect Auestions		False Confidence Justification		Report Generation	
			*acc*_*h*_	*acc*_*b*_	*acc*_*h*_	*acc*_*b*_	*acc*_*h*_	*acc*_*b*_	*acc*_*h*_	*acc*_*b*_	*acc*_*h*_	*acc*_*b*_	CHAIR	F_1_
General models	GPT-4 V	Commercial	0.324	0.471	**0.978**	0.244	0.244	0.262	**0.356**	0.186	0.366	0.378	0.665	0.338
	LLaVa	13B	0.044	0.500	0.014	0.344	**0.478**	0.280	0.020	0.366	0.250	0.360	0.760	0.194
	MiniGPT	7B	0.228	0.508	0.024	0.326	0.108	0.124	0.006	0.030	**0.490**	0.326	0.938	0.040
Medical models	LLaVa-Med	7B	0.170	0.457	0.110	0.216	0.028	0.164	0.004	0.168	0.172	0.244	0.737	0.218
	Med-Flamingo	8B	–	–	–	–	–	–	–	–	–	–	0.831	0.133
CXR models	LLM-CXR	3B	0.348	0.472	0.104	0.220	0.094	0.130	0.046	0.244	0.220	0.256	0.570	0.401
	XrayGPT	7B	0.176	0.173	0.164	0.286	0.154	0.140	0.016	0.030	0.230	0.132	0.576	0.278
	CheXagent	8B	**0.378**	**0.526**	0.154	**0.410**	0.258	0.458	0.182	**0.540**	0.094	**0.462**	**0.461**	**0.506**

**Table 3.** Performance on false confidence justification. We suggest the incorrect answer to the model in the first two columns. For baselines, we do not suggest an answer in the prompt in the last column. We also try suggesting the correct answer to the model in the middle two columns.

LVLM	Wrong suggested answer		Correct suggested answer		No suggested answer
	*r*_*disagree*_	*r*_*correct*_	*r*_*disagree*_	*r*_*correct*_	*r*_*baseline*_
GPT-4 V	0.746	0.366	0.534	0.466	0.378
LLaVa	0.562	0.250	0.504	0.496	0.360
MiniGPT	0.938	0.490	0.950	0.050	0.326
LLaVa-Med	0.308	0.172	0.540	0.460	0.244
LLM-CXR	0.376	0.220	0.310	0.690	0.256
CheXagent	0.964	0.094	0.768	0.232	0.462

## Data Availability

Our curated MedVH dataset is available at PhysioNet as https://physionet.org/content/medvh. Our source code is available at GitHub as https://github.com/dongzizhu/MedVH.
