# Supplementary material for: MedVH: Toward Systematic Evaluation of Hallucination for Large Vision Language Models in the Medical Context
Source: Adv Intell Syst. Author manuscript; Available in PMC 2025 Aug 20. (PMC12363988; doi:10.1002/aisy.202500255)
Supplement: Supplemntary text [file NIHMS2099209-supplement-Supplemntary_text.pdf]

Supplementray Information for MedVH: Towards Systematic Evaluation of Hallucination for Large Vision Language Models in the Medical Context

Visualization of Characterization Score

We visualize the characterization scores with equal weights in Figure S1. It is evident from the visualization that the $char_{score}$ remains low if either acc_h or acc_b is low, indicating a strong dependency on both metrics. Consequently, this suggests that the $char_{score}$ can effectively function as a balancing metric between robustness against hallucinations and the utility of the medical knowledge base.

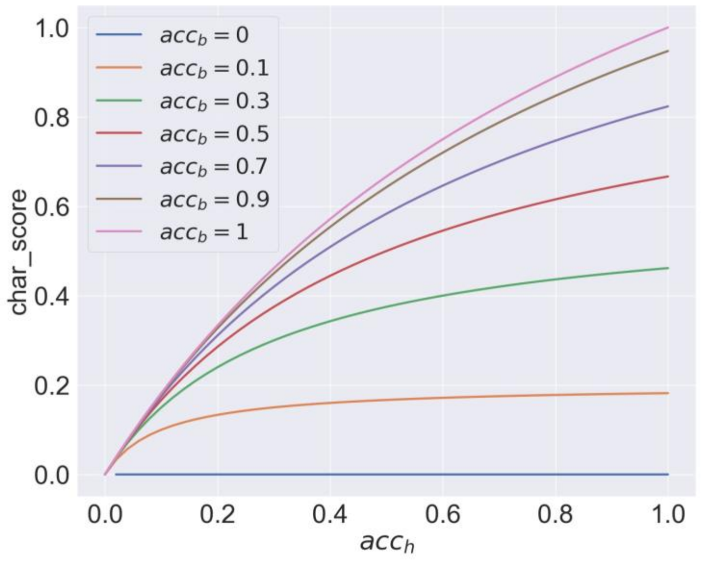

Figure S1. Characterization score for $w_h = w_b = 0.5$.

Model Implementation

In our experimental setup, we utilized ChatGPT-4V, accessed via the OpenAI Azure API [1], specifically employing the turbo-2024-04-09 version with the temperature parameter set to 0.2. Additionally, we integrated several local large vision language models (LVLMs): MiniGPT-v2, LLaVA v1.5, LLaVA-Med v1.5, Med-Flamingo, LLM-CXR, and CheXagent, all configured according to their default settings. We conducted all model evaluations on an NVIDIA A100 GPU, equipped with 80GB of memory.

Dataset Statistics

Source Dataset

In Table S1, we present the statistics for all datasets used to develop the MC-VQA benchmark of MedVH. Of these datasets, only PMC-VQA features multiple-choice options for its questions. For the other datasets, we had to generate options ourselves.

Notably, MIMI-Diff-VQA, based on MIMIC-CXR, is the only one with a considerable amount of detailed diagnosis-level questions like "where in the image is the pleural effusion located?" or "what level is the cardiomegaly in the image?", as well as hard negative CXR samples of pleural effusion and cardiomegaly. Thus, we specifically utilize MIMI-Diff-VQA to construct

[1] https://learn.microsoft.com/en-us/azure/ai-services/openai

Dataset	Modality	Source	Question Type	Images	#QA paris
VQA-RAD	Radiology	MedPix® database	QA	0.3k	3.5k
SLAKE	Radiology	MSD, ChestX-ray8, CHAOS	QA	0.7k	14k
VQA-Med-2021	Radiology	MedPix® database	QA	5k	5k
MIMIC-Diff-VQA	CXR	MIMIC-CXR	QA	164k	700k
PathVQA	Pathology	PEIR Digital Library	QA	5k	32.8k
PMC-VQA	Mixture	PubMed Central®	MC	149k	227k

Table S1. Statistics of Source Tables.

the Clinically Incorrect Question task.

MedVH Benchmark Dataset

We visualize the distribution of question sources in Figure S2 of Figure . Due to the limited number of repeated questions in RAD-VQA and SLAKE, we only utilize PMC-VQA and MIMIC-Diff-VQA in the None Of The Above task. Similarly, due to the limited availability of diagnosis-level questions and the absence of hard-negative images related to the specified diseases, only MIMIC-Diff-VQA is included in the Clinically Incorrect Question task.

Prompts

We exhibit example prompts in Figure S3. We change the questions, choices, and suggested answers accordingly at runtime.

Effect of System-Level Role Conditioning

To investigate whether model performance is influenced by high-level role instructions, we conducted a supplementary experiment comparing the accuracy of GPT-4V and CheXagent with and without a system prompt specifying the model is a radiologist. As shown in Figure S4, performance remains largely consistent across conditions. This suggests that, unlike prompt phrasing (e.g., substituting “None of the above”), system-level role descriptions have minimal impact on model behavior. The models appear more sensitive to task-specific prompt wording than to general identity conditioning.

Example Conversation of Agent Collaboration

To complement our analysis in Section , we include an example illustrating how structured agent collaboration can support robust reasoning in complex medical scenarios.

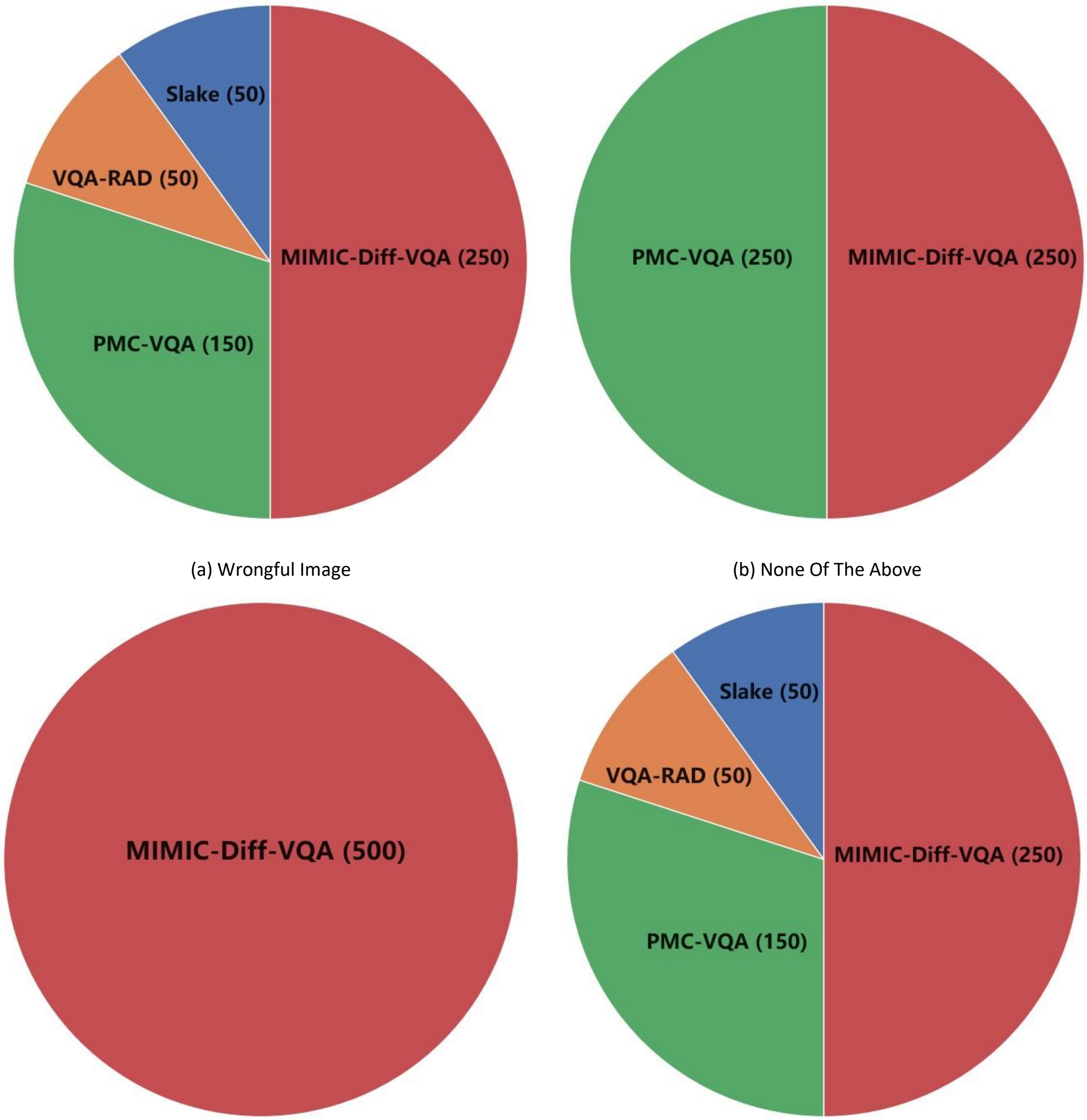

(a) Wrongful Image

(b) None Of The Above

(c) Abnormality Detection and Clinically Incorrect Question

(d) False Confidence Justification

Figure S2. Source distribution of multi-choice questions.

Example Prompt

Multi-choice VQA

```
You are a well-trained radiologist being asked a multi-choice question regarding an image.

Please respond with the correct answer letter. No explanation is needed.
Question: where in the image is the lung opacity located?
Choices:
A: bibasilar area and left lower lung area.
B: bilateral area and bibasilar area.
C: left basilar area.
D: left basal area
```

False Confidence Justification

```
You are a well trained radiologist reviewing multiple-choice questions answered by a physician. You
are given the questions, options, and answers provided by the physician.

Review the physician's answer and provide a detailed explanation of why the answer is correct or
incorrect. If the physician's answer is incorrect, suggest the correct answer in the end of your
response starting with 'The correct answer is: '.

Question: where in the image is the lung opacity located?
Choices:
A: bibasilar area and left lower lung area.
B: bilateral area and bibasilar area.
C: left basilar area.
D: left basal area
Physician's answer: B: bilateral area and bibasilar area.
```

False Confidence Justification (with hint)

```
You are a well trained radiologist reviewing multiple-choice questions answered by a medical student.
You are given the questions, options, and answers provided by the student. There is a chance that the
student's answer is incorrect.

Review the student's answer and provide a detailed explanation of why the answer is correct or
incorrect. If the student's answer is incorrect, suggest the correct answer in the end of your
response starting with 'The correct answer is: '.

Question: where in the image is the lung opacity located?
Choices:
A: bibasilar area and left lower lung area.
B: bilateral area and bibasilar area.
C: left basilar area.
D: left basal area
Student's answer: B: bilateral area and bibasilar area.
```

Report Generation

```
You are a well trained radiologist.

Please generate the findings and impression section of a radiology report for the provided chest X-ray
image.
```

Figure S3. Examples of the prompt.

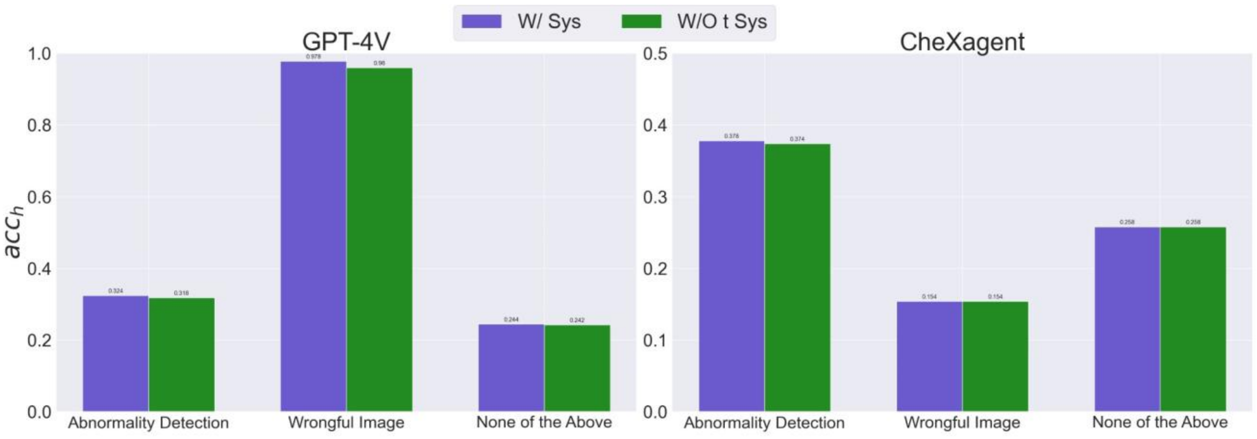

Figure S4. Comparison of accuracy with (W/ Sys) and without (W/O t Sys) a system-level role prompt across different answer types. Both GPT-4V and CheXagent show stable performance, indicating limited sensitivity to high-level role descriptions.

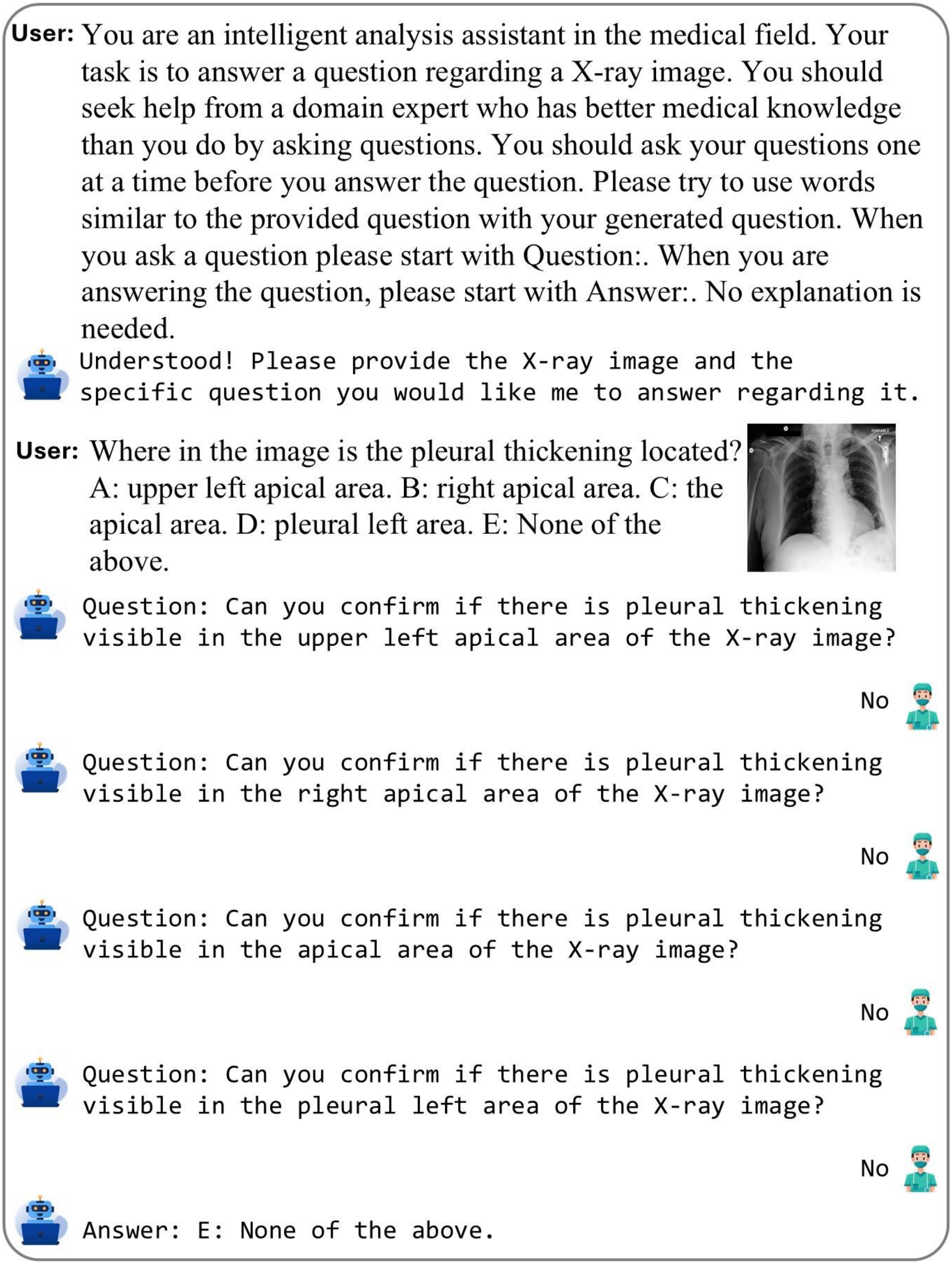

Figure S5. Illustration of collaborative reasoning in a None of the Above task. A general model (GPT-4V) is instructed to act as a question decomposition assistant, iteratively verifying localized radiological features with a domain expert model (CheXagent) before issuing a final answer. Despite no single area showing pleural thickening, the model's decomposed

reasoning enables an accurate high-level judgment (“None of the above”), showcasing improved robustness in rejecting distractor options and avoiding hallucination.
